# Statistical Mechanics and Thermodynamics of Liquids and Crystals

**DOI:** 10.3390/e23060715

**Published:** 2021-06-04

**Authors:** Santi Prestipino

**Affiliations:** Dipartimento di Scienze Matematiche ed Informatiche, Scienze Fisiche e Scienze della Terra, Università degli Studi di Messina, Viale F. Stagno d’Alcontres 31, 98166 Messina, Italy; sprestipino@unime.it

Thermodynamic phases are the most prominent manifestation of emergent behavior. Among them, crystals and liquids traditionally epitomize the antagonistic concepts of order and disorder (i.e., the presence or absence of a symmetry). According to common wisdom, the competition for stability between solid and liquid reflects the struggle between the opposite tendencies of energy minimization and entropy maximization, which is regulated by temperature. It goes without saying that the previous statement is in fact too simplistic, since in equilibrium the guiding principle is rather the minimization of the thermodynamic potential (maximization of the Massieu function) appropriate to the given control parameters. For instance, crystallization sometimes occurs with the purpose to maximize entropy (as for hard spheres under pressure). A further example is provided by superfluid, which can be one of the phases of minimum energy/enthalpy at zero temperature.

The relevance of the solid/liquid dichotomy for statistical physics cannot simply be overstated. It was under the pressure of accurately locating the solid–liquid transition in simple-fluid models that “exact” free-energy methods were initially developed [1,2]. Since then, variations on these methods have been employed to compute the phase diagram of many complex fluids, such as liquid crystals [3], cluster crystals [4], and fluids of patchy particles [5]. Generally speaking, the full control of phase behavior can help in the synthesis of artificial materials with the desired specifications.

Turning to theory, while the exact determination of the partition function for a non-trivial system with solid, liquid, and vapor phases will probably never be accomplished, there are nevertheless variational treatments of the solid–liquid transition (density functional theories) that have by now reached a high degree of sophistication (see, e.g., Refs. [6,7]). However, a schematic phase diagram with the standard three phases can be obtained with less effort, see for instance the mean-field analysis of the Potts lattice gas [8].

It is usually thought that (classical) liquid is a unique phase, while crystals are a multitude. In fact, this is only partially true, since liquids composed of hydrogen-bonded molecules exhibit a number of so-called water-like “anomalies” that make them different in many respects from conventional (rare-gas) fluids (see, e.g., [9]). The relationship between such anomalies and solid polymorphism/polyamorphism is an important topic of statistical physics. Phase diagrams with many solid polymorphs are the rule for simple substances (e.g., Na [10]) under huge pressure. Here, exact free-energy methods face a serious limitation, since the crystalline structures—Bravais and non-Bravais—being potentially relevant are countless. Metadynamics [11] and evolutionary algorithms [12] are a possible way out, since they do not involve any assumption on the topology of the energy landscape.

Condensed-matter physics also features a wide variety of phases with mixed solid and liquid characteristics. Hexatic fluids [13], liquid-crystal smectics [14], crystalline membranes [15], and quantum supersolids [16] are just a few examples of hybrid states of aggregation, with others yet to be discovered. In the last few years, a new category of systems, i.e., self-assembling materials, has fallen under the scrutiny of the statistical-physics community (see, e.g., [17]). Here, some kind of order at the mesoscopic scale spontaneously emerges from the interaction between simple microscopic units. For these systems, the precise interplay between thermodynamics and kinetics in the onset of aggregates is still being worked out. As we move forward in the bottom-up investigation of biological matter, the concerted role of energy and entropy in the formation of structures with hierarchical order will be made more clear.

This Special Issue collects articles published between April 2020 and May 2021, highlighting novel results in the application of statistical thermodynamics to liquids and crystals.

In the first of these articles [18], the focus is on the virial equation of state for mixtures of hard hyperspheres. While such systems obviously have no counterpart in the real world, they still represent a useful playground where to explore the effectiveness of approximations routinely employed in three dimensions. If geometric considerations play a leading role in the crossover from three to five dimensions, then simple analytic extensions of approximations that proved successful in lower dimensions would provide accurate equations of state for hard hyperspheres in the fluid phase. It turns out that the sole requirement of reproducing the exact second and third virial coefficients yields, in four and five dimensions, approximate equations of state of overall good quality in the comparison with computer-simulation data.

Moderately dense particles driven far from equilibrium are much harder to attack theoretically. In this case, numerical simulation is the only method to assess the accuracy of fluid dynamic equations. The second article in this Special Issue [19] is an attempt to investigate turbulent convection using the Boussinesq approximation—accounting for the variation of density with temperature only in the buoyancy term of the Navier–Stokes equation. The approach of numerical simulation allows one to analyze both heat and mass flow under a variety of boundary conditions, with potential applications in oceanography, geophysics, astrophysics, and industry.

A well-established approach to the entropy of a simple fluid is the so-called multiparticle correlation expansion (MPCE), expressing the statistical entropy as a sum of contributions from increasingly large numbers of particles. Upon truncating the MPCE after the two-body term $S_{2}$, one has an estimate of the exact entropy that turns out to be accurate right at the point of transition into a crystal, leading to a freezing criterion [20] that has had a certain success in the past. After reviewing the history of the entropy MPCE, Ref. [21] inquires into the possibility of formulating an analogous entropic criterion for melting, given that a MPCE formally holds also for the entropy of a crystal. However, the computation of $S_{2}$ proves to be a formidable task even for a crystal of hard disks, thus dampening the enthusiasm for any melting criterion based on the numerical evaluation of the two-body entropy.

Reference [22] investigates pattern formation in a two-dimensional lattice gas system. Lattice gases allow for an exact thermodynamic analysis at zero temperature, since all possible ground states can be enumerated and compared with each other in terms of enthalpy. Such studies can be helpful to design and control the functionalization of colloidal particles with polymers.

Hard-core bosons are the quantum counterpart of lattice-gas particles. Lattice systems of bosonic particles provide models where the competition between itinerant and localized quantum states can be examined in full detail. The prototype of all such models is the celebrated Bose-Hubbard model, describing the behavior of ultracold bosonic atoms trapped in an optical lattice. Upon increasing the intensity of laser light, the confining potential gets deeper until a transition occurs from superfluid to Mott insulator (i.e., a normal cluster solid) [23,24]. The Bose–Hubbard model and its variants provide an ideal setting for exploring strong-correlation effects in quantum systems, which now are also studied for bosons on the nodes of a spherical mesh [25,26]. However weird this geometry may seem, traps located at the vertices of a polyhedron can be fabricated with optical tweezers and loaded with Rydberg atoms [27]. In particular, a system of bosons in a cubic mesh [25] offers the opportunity to assess the validity of mean-field theory, as well as to uncover the manifestations of superfluidity in a small quantum system.

Spatial correlations between triplets of particles in a fluid or solid are notoriously difficult to investigate in full extent, owing to heavy CPU-time and memory requirements. Yet, triplet correlations convey crucial information for any statistical analysis aiming to go beyond the traditional pairwise approximation. Reference [28] investigates triplet correlations in a fluid of quantum hard spheres, as well as in two crystalline phases of the same system. Using path-integral Monte Carlo simulations, the author of [28] delves into the accuracy of a few closure relations expressing the triplet distribution function in terms of two-body terms, eventually identifying a combination of closures that performs well in rather disparate conditions.

The emergency caused by the COVID-19 pandemic has boosted a large amount of research activities in the last year with the purpose of clarifying the many open questions that arise in connection with the transmission of the infection. Our Special Issue too contains an article on the problem [29], about diffusion in the air of liquid nanodroplets containing the infective agent. Using a molecular theory, the author of [29] derives an effective Hamiltonian for gas atoms and liquid droplets which accounts for the interaction and correlation effects induced by the granular structure of the droplets. Similar theoretical studies may be viewed as complementary to atomistic simulations, which are obviously much more computationally demanding.

The last article in this Special Issue [30] deals with a numerical investigation of the self-assembling behavior of a system made up of asymmetric dimers and marbles (“disks”) confined in a spherical surface, as is realized by, e.g., a mixture of colloidal particles spread over the surface of an oil droplet. In the model, the formation of disk aggregates is triggered by a short-range attraction between the disk and one of the monomers. For low disk compositions, only small clusters are found, while for higher composition values, we observe the onset of long flexible chains which, at sufficiently high density, give origin to an intricate network on the sphere. When disks are much larger than dimers, square-ordered patches are formed instead, similar to the truncated triangular crystals of polystyrene spheres growing on the inside walls of water droplets [31], and in striking contrast to the spanning triangular crystal, punctuated by islands of defects, that is promoted by entropy alone in dense hard disks on a sphere [32].

It is our hope that this Special Issue leaves the reader with the impression that the field of liquids and crystals is a vivid research area, full of problems still waiting for solution, and open to surprises.
